# Multi-molecular imaging showing tumour heterogeneity and differing diagnostic performance in a case with metastatic pheochromocytoma

**DOI:** 10.1186/s41824-025-00267-3

**Published:** 2025-09-01

**Authors:** Fredrik Hedeer, Jenny Oddstig, Páll Hallgrimson, Katarina Fagher

**Affiliations:** 1 Department of Clinical Physiology and Nuclear Medicine, Lund University, Skåne University Hospital, Entrégatan 7, Lund, S-221 85 Sweden; 2 Department of Medical Radiation Physics, Lund University, Skåne University Hospital, Lund, Sweden; 3 Department of Surgery, Lund University, Skåne University Hospital, Lund, Sweden; 4 Department of Endocrinology, Lund University, Skåne University Hospital, Lund, Sweden

**Keywords:** PET, Scintigraphy, Molecular imaging, ^18^F-mFBG, ^18^F-DOPA, ^68^Ga-DOTA-TOC, ^123^I-mIBG, Pheochromocytoma, Paraganglioma, Neuroendocrine tumour

## Abstract

A patient with multiple endocrine neoplasia 2A (MEN2A) and a suspicion of metastasized pheochromocytoma, underwent multi-molecular positron emission tomography (PET) imaging with the recently presented radiopharmaceutical [^18^F]-metafluorobenzylguanidine (mFBG), [^68^Ga]Ga-DOTA-TOC and [^18^F]F-dihydroxyphenylalanine (DOPA) as well as scintigraphy with [^123^I]-metaiodobenzylguanidine (mIBG). The methods showed different diagnostic performance, with the highest number of suspected malignant lesions detected by [^18^F]-mFBG-PET, and revealed insights into tumour heterogeneity.

## Introduction

Pheochromocytomas are neuroendocrine tumours originating from chromaffin cells of the adrenal medulla (Mete et al. 2022; Nölting et al. 2022). They are related to paraganglioma and are also referred to as adrenal paraganglioma. Although many pheochromocytomas are sporadic, a substantial number of cases is associated with germline mutations in various susceptibility genes (Nölting et al. 2022). Pheochromocytomas exhibit specific properties that can be targeted in diagnostic imaging with different molecular nuclear imaging methods (Mete et al. 2022; Nölting et al. 2022; Taïeb et al. 2019). As many other tumours of neuroendocrine origin, pheochromocytomas overexpress somatostatin receptors (SSTR), which can serve as targets for imaging as well as therapy (Patel et al. 2021; Reubi et al. 1992). Positron emission tomography (PET) with ^68^Ga-labeled SSTR analogs has evolved as a corner stone in diagnostic imaging of neuroendocrine tumours, including pheochromocytomas (Taïeb et al. 2019; Patel et al. 2021). Another specific quality of pheochromocytomas is the synthetization and secretion of hormones, mainly catecholamines such as epinephrine and norepinephrine. In the synthetization of catecholamines, pheochromocytoma cells decarboxylate amino acids such as dihydroxyphenylalanine (DOPA). DOPA is taken up through a membrane protein, L-type amino acid transporter (LAT), primarily LAT1 (Taïeb et al. 2019). Labeling DOPA with fluorine-18 ([^18^F]F-DOPA), this mechanism can be targeted with PET imaging. Furthermore, the catecholamine turnover can be targeted using a norepinephrine analog which is taken up by the pheochromocytoma cells through a norepinephrine transporter (NET) protein. The traditional way to image this mechanism is scintigraphy with an iodine-123 labeled norepinephrine analog ([^123^I]-metaiodobenzylguanidine (mIBG) (Taïeb et al. 2019). Recently, a norepinephrine analog molecule identical to mIBG, labeled with fluorine-18, has been presented ([^18^F]-metafluorobenzylguanidine (mFBG), allowing for imaging of the same pathophysiological mechanism with PET, with potentially superior diagnostic accuracy compared to scintigraphy (Pandit-Taskar et al. 2018; Pauwels et al. 2023). However, comparison of the diagnostic accuracy of this new PET tracer to the above-mentioned established PET tracers are lacking.

According to guidelines, the diagnostic molecular imaging method of choice is dependent on presence of underlying mutations as well as tumour origin (Nölting et al. 2022; Taïeb et al. 2019). However, published studies comparing the diagnostic accuracy between methods in specific patient groups are based upon small sample sizes, why more studies are needed.

We present a patient case with multiple endocrine neoplasia 2A (MEN2A) and suspected recurrence of pheochromocytoma, who underwent diagnostic molecular imaging with several nuclear imaging methods targeting different disease specific properties.

## Case report

A 40-year-old female with MEN2A had previously undergone total thyroidectomy for medullary thyroid cancer (MTC) in 1995, total left adrenalectomy for pheochromocytoma in 2007 and cortical-sparing right adrenalectomy for pheochromocytoma in 2020. In 2024, rising plasma levels of metanephrines (methoxy adrenaline 0.62 nmol/L (reference < 0.3) and methoxy noradrenaline 1.6 nmol/L (reference < 0.8)), prompted molecular imaging to assess recurrence of pheochromocytoma. Serum calcitonin levels were normal. The patient underwent SSTR-PET/computed tomography (CT) with [^68^Ga]Ga-DOTA-TOC, [^18^F]F-DOPA-PET/CT, [^123^I]-mIBG-scintigraphy and PET/CT with the new radiopharmaceutical [^18^F]-mFBG, on separate occasions with 99 days between the first (SSTR-PET/CT) and the last ([^123^I]-mIBG-scintigraphy) examination. The patient has given informed consent to this publication.

## Methods

Imaging was performed according to clinical routine protocols. For PET/CT, 127 MBq of [^68^Ga]Ga-DOTA-TOC, 254 MBq of [^18^F]F-DOPA and 256 MBq of [^18^F]-mFBG, respectively, was injected intravenously. PET/CT images from mid thighs to the base of scull were acquired on a GE Discovery MI PET/CT scanner after 60 min of accumulation time for all radiopharmaceuticals. CT attenuation corrected images were reconstructed with a block-sequential regularization-expectation-maximization reconstruction algorithm (beta-value: 900 for [^68^Ga]Ga-DOTA-TOC-PET, 700 for [^18^F]F-DOPA-PET and 550 for [^18^F]-mFBG-PET), using time-of-flight (TOF), point spread function modelling and standard corrections for scatter, dead time and physical decay. For [^123^I]-mIBG-scintigraphy, the patient received potassium iodide for thyroid blocking according to standard schedule. 354 MBq of [^123^I]-mIBG was injected intravenously. Planar whole-body images and SPECT/CT images of the thorax and abdomen were acquired after 24 h of accumulation time on a GE Discovery NM/CT 670 gamma camera, using an extended low-energy general purpose (ELEGP) collimator (planar scanning velocity: 4.8 cm/min). CT attenuation corrected SPECT images were reconstructed using Ordered Subset Expectation Maximization (OSEM) with resolution recovery, 4 iterations, 10 subsets, a Butterworth postfilter, and standard correction for scatter.

## Results

**Fig. 1:**
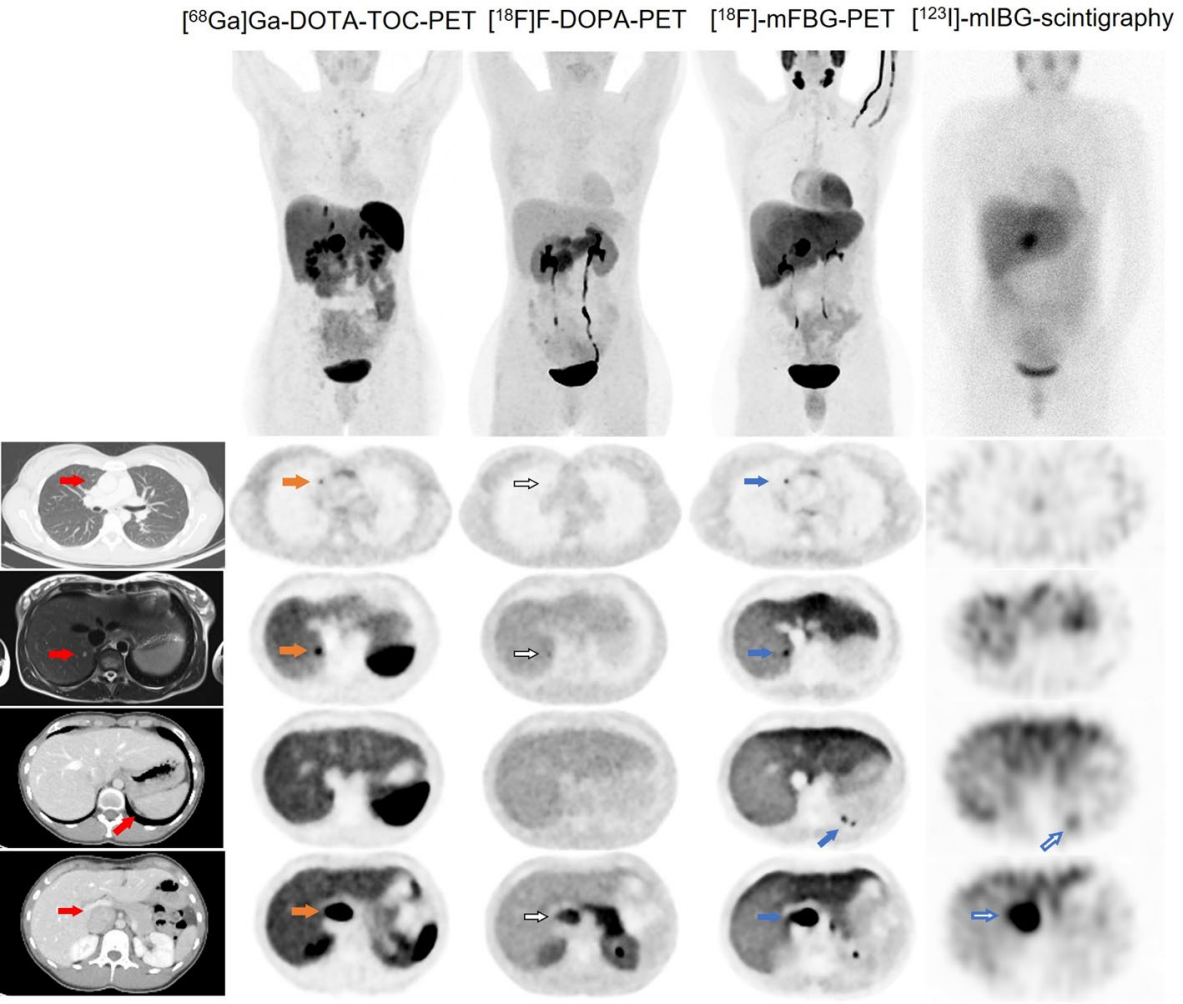
Four molecular imaging modalities and corresponding morphologic imaging in a case with suspected metastatic pheochromocytoma. Columns display different imaging methods: Column 1– morphologic images with CT or MRI (red arrows), Column 2– somatostatinreceptor-PET with [^68^Ga]Ga-DOTA-TOC (orange arrows), Column 3– [^18^F]F-DOPA-PET (open black arrows), Column 4– [^18^F]-mFBG-PET (blue arrows) and Column 5– [^123^I]-mIBG-scintigraphy (open blue arrows). Row 1 shows maximum intensity projections and a whole-body planar image, respectively, and rows 2–5 transversal images in different planes. Row 2 shows a suspected pulmonary metastasis with uptake on [^68^Ga]Ga-DOTA-TOC-PET and [^18^F]-mFBG-PET, relatively lower uptake and hardly detectable on [^18^F]F-DOPA-PET and no uptake on [^123^I]-mIBG-scintigraphy. Row 3 shows a suspected liver metastasis with uptake on [^68^Ga]Ga-DOTA-TOC-PET and [^18^F]-mFBG-PET, relatively lower uptake and hardly detectable on [^18^F]F-DOPA-PET and no uptake on [^123^I]-mIBG-scintigraphy. Row 4 shows two suspected metastases between the spleen and diaphragm with uptake on [^18^F]-mFBG-PET and [^123^I]-mIBG-scintigraphy but no uptake on [^68^Ga]Ga-DOTA-TOC-PET or [^18^F]F-DOPA-PET. Row 5 shows a portocaval suspected malignant lesion with uptake on all imaging modalities

Images of all four diagnostic methods are shown in Fig. 1. Altogether, lesions with uptake and morphological correlates, suspected of metastases, were shown in the left supraclavicular fossa, in the right lung, between the spleen and diaphragm, in the liver and in a portocaval lesion. Additional pulmonary nodules (2–4 mm in diameter) were found on CT with no uptake on any of the molecular imaging methods. As shown in Fig. 1, the different suspected metastatic lesions showed various detectability between imaging methods. Furthermore, intensity of radiopharmaceutical uptake in detectable suspected metastatic lesions, varied both within and between imaging methods. The highest number of lesions was detected by [^18^F]-mFBG-PET, followed by [^68^Ga]Ga-DOTA-TOC-PET, [^18^F]F-DOPA-PET and lastly the lowest number by [^123^I]-mIBG-scintigraphy.

Surgery of the largest suspected malignant lesion, the portocaval lesion, was performed for diagnosis and tumor debulking. Histopathology showed a cell rich tumour, surrounded by normal adrenal tissue and with immunohistochemistry positive for chromogranin A, consistent with pheochromocytoma, probably emanating from the remains of the previously operated right adrenal. The remains of the previously operated right adrenal were also surgically removed, and histopathology showed normal adrenal tissue. None of the other suspected metastatic lesions were surgically removed or histopathologically verified. After surgery, plasma levels of metanephrines decreased (methoxy adrenaline 0.12 nmol/L (reference < 0.3) and methoxy noradrenaline 0.17 nmol/L (reference < 0.8)).

## Discussion

We present a patient case with MEN2A who underwent multi-modality molecular diagnostic imaging for suspected recurrence of pheochromocytoma. Imaging revealed multiple lesions suspected of metastases, however with diverging lesion detectability within and between the different molecular imaging methods. The highest number of lesions suspected of metastases was detected by [^18^F]-mFBG-PET.

There may be several potential explanations for the differences in lesion detectability found between the four different molecular imaging methods. The methods target three different properties that can be expressed in chromaffin/pheochromocytoma cells– somatostatinreceptor expression ([^68^Ga]Ga-DOTA-TOC-PET, presence of LAT1 membrane proteins ([^18^F]F-DOPA-PET) and catecholamine turnover through presence of NET membrane proteins ([^18^F]-mFBG-PET and [^123^I]-mIBG-scintigraphy). Although categorized as pheochromocytoma, tumours in individual patients may exhibit differences in specific properties that may manifest in diagnostic molecular imaging. To date, studies comparing diagnostic accuracy between molecular imaging methods, considers SSTR-PET to be the first line diagnostic method for pheochromocytoma/paraganglioma patients carrying *SDHx* related mutations and most often for paraganglioma originating in the head and neck region, and [^18^F]F-DOPA-PET to be the first line diagnostic method for pheochromocytoma/paraganglioma patients carrying other mutations (such as MEN2A) and for tumours of adrenal origin including sporadic cases (Nölting et al. 2022; Taïeb et al. 2019; Jha et al. 2022; Archier et al. 2016; Janssen et al. 2016a, b). In the presented patient case, [^18^F]F-DOPA-PET may be considered the first line diagnostic method according to guidelines. However, in this case, lesions suspected to be metastases were more distinctly visualized with SSTR-PET than [^18^F]F-DOPA-PET. This highlights the complexity of these tumours and suggests that group-level recommendations may not always be applicable to individual patients. Furthermore, it is important to note that studies comparing the diagnostic accuracy of different molecular imaging methods, most often include a limited number of patients given the rarity of pheochromocytomas and paragangliomas. Thus, more studies are needed to gain more insights in how to use different molecular imaging methods in individual patients. To the best of our knowledge, there are currently no previous studies comparing [^18^F]-mFBG-PET with other PET tracers in these patient groups. [^123^I]-mIBG-scintigraphy has long been used for diagnostic imaging in pheochromocytoma/paraganglioma patients. However, limited diagnostic performance and more widespread use of PET/CT with specific tracers, have ended up in using [^123^I]-mIBG-scintigraphy mainly for selection of potential patients to be candidates for [^131^I]-mIBG therapy (Taïeb et al. 2019). In this patient case, [^123^I]-mIBG-scintigraphy detected the least number of suspected malignant lesions. In contrast, [^18^F]-mFBG-PET detected the highest number of suspected malignant lesions of the four imaging modalities, despite targeting the same molecular structure as [^123^I]-mIBG-scintigraphy. Both tracers share the same molecular structure but differ in their radioactive labels, iodine-123 for [^123^I]-mIBG and fluorine-18 for [^18^F]-mFBG (Garg et al. 1994). Studies comparing [^123^I]-mIBG-scintigraphy and [^18^F]-mFBG-PET in patients with tumours originating from chromaffin cells, have shown a superior diagnostic performance of [^18^F]-mFBG-PET (Pandit-Taskar et al. 2018; Pauwels et al. 2023; Borgwardt et al. 2024). Although, specific tumour cell uptake of [^18^F]-mFBG seems to be lower than [^123^I]-mIBG in vitro, [^18^F]-mFBG tumour cell uptake in vivo seems to be higher (Zhang et al. 2014a, b). The overall distribution of [^18^F]-mFBG and [^123^I]-mIBG seems to be similar in vivo (Pandit-Taskar et al. 2018; Pauwels et al. 2023; Borgwardt et al. 2024). Altogether, differences in diagnostic performance between [^18^F]-mFBG-PET compared to [^123^I]-mIBG-scintigraphy are most probably a result of the higher sensitivity and resolution in PET imaging compared to gamma camera imaging (Pandit-Taskar et al. 2018; Pauwels et al. 2023; Borgwardt et al. 2024). To the best of our knowledge, this is the first patient case comparing the diagnostic performance of [^18^F]-mFBG-PET with other PET tracers. Further studies in larger patient cohorts are needed to evaluate the diagnostic performance of [^18^F]-mFBG-PET compared to the established PET tracers used in pheochromocytoma/paraganglioma patients. As previously stated, [^123^I]-mIBG scintigraphy is currently recommended mainly for selection of patients for isotope therapy and not primarily for diagnostic imaging. However, based on this patient case and currently published studies comparing the diagnostic performance of [^18^F]-mFBG-PET vs. [^123^I]-mIBG-scintigraphy, imaging catecholamine turnover by targeting the NET membrane protein with [^18^F]-mFBG-PET, seems to be a promising molecular target also for diagnostic imaging.

Another explanation of differences in detectability of different suspected metastatic lesions within and between diagnostic molecular imaging methods, may be that metastases from the same tumour origin may exhibit various differentiation. Thus, different pheochromocytoma/paraganglioma tumour lesions may evolve different expression of somatostatin receptors, LAT1 and NET transporter proteins, which then potentially could manifest in variations in detectability using molecular imaging methods targeting these specific properties. Furthermore, an individual patient may have tumours and/or metastases of different origin that may or may not be detectable by a molecular imaging method with a specific tracer. This patient case with MEN2A, also had a medical history of MTC. Thus, hypothetically, differences in lesion detectability within and between the molecular imaging methods could be due to different tumour origin of the different lesions. The preferred diagnostic molecular imaging method to be used in patients with MTC is considered to be [^18^F]F-DOPA-PET (Treglia et al. 2023). In this case, [^18^F]-mFBG-PET and [^68^Ga]Ga-DOTA-TOC-PET identified a higher number of suspected malignant lesions than [^18^F]F-DOPA-PET. All lesions detected by [^18^F]F-DOPA-PET were also visualized by both [^18^F]-mFBG-PET and [^68^Ga]Ga-DOTA-TOC-PET with no additional lesions seen exclusively on [^18^F]F-DOPA-PET. Furthermore, patient serum levels of calcitonin were normal. Lastly, some lesions, such as the pulmonary nodules detected on CT, may have been too small to be detectable with the molecular imaging methods, or they may represent non-specific benign lesions.

A limitation in this patient case was that not all detected lesions on imaging were histopathologically verified as tumours/metastases. Thus, although the lesions found on both morphological and functional imaging modalities are highly suspected for metastases, this has not been confirmed. Another limitation was the time elapsing between the first and the last examination. However, on CT there were no morphologic changes of the lesions between the first and the last examination. Current plans for patient follow-up are biochemical surveillance and repeated imaging.

To conclude, in this patient case with MEN2A and a suspicion of pheochromocytoma recurrence, lesions suspected of metastases showed different detectability by four different molecular imaging methods, where the highest number of suspected metastatic lesions were detected by [^18^F]-mFBG-PET. Although imaging guidelines have recommendations of first line diagnostic methods in pheochromocytoma/paraganglioma patients based on presence of specific gene mutations and tumour origin, this case shows that this may vary in individual patients, and probably should be accounted for in this era of individualized/personalized medicine. The differences between the imaging modalities may have significant clinical implications for staging and follow-up in these kinds of patients. Future studies are needed to determine whether the use of multiple molecular imaging methods may add extra diagnostic value in some patient populations, as well as to explore the potential prognostic and therapeutic implications of multi-molecular imaging approaches. Furthermore, the newly developed PET tracer [^18^F]-mFBG seems to be a promising diagnostic molecular imaging method in patients with pheochromocytoma/paraganglioma. Future studies will have to explore the clinical usefulness of this method in specific patient groups, including comparison with molecular imaging methods with other specific tracers.

## Data Availability

Not applicable.
